# Association of epidural analgesia during labor with neurodevelopment of children during the first three years: the Japan Environment and Children’s Study

**DOI:** 10.1265/ehpm.22-00088

**Published:** 2022-09-28

**Authors:** Masayuki Shima, Narumi Tokuda, Hideki Hasunuma, Yoshiko Kobayashi, Hiroyuki Tanaka, Hideaki Sawai, Hiroaki Shibahara, Yasuhiro Takeshima, Munetaka Hirose, the Japan Environment and Children’s Study (JECS) Group

**Affiliations:** 1 Hyogo Regional Center for the Japan Environment and Children’s Study, Hyogo Medical University, Nishinomiya, Japan; 2 Department of Public Health, School of Medicine, Hyogo Medical University, Nishinomiya, Japan; 3 Department of Anesthesiology and Pain Medicine, School of Medicine, Hyogo Medical University, Nishinomiya, Japan; 4 Department of Obstetrics and Gynecology, School of Medicine, Hyogo Medical University, Nishinomiya, Japan; 5 Department of Pediatrics, School of Medicine, Hyogo Medical University, Nishinomiya, Japan

**Keywords:** Birth cohort study, Children, Epidural analgesia during labor, Japan Environment and Children’s Study (JECS), Maternal age, Neurodevelopment, the Ages and Stages Questionnaires, Third Edition (ASQ-3)

## Abstract

**Background:**

Epidural analgesia relives pain during labor. However, the long-term effects on neurodevelopment in children remain unclear. We explored associations between exposure to epidural analgesia during labor and childhood neurodevelopment during the first 3 years of life, in the Japan Environment and Children’s Study (JECS), a large-scale birth cohort study.

**Methods:**

Pregnant women were recruited between January 2011 and March 2014, and 100,304 live births of singleton children born at full-term by vaginal delivery, and without congenital diseases were analyzed. Data on mothers and children were collected using a self-administered questionnaires and medical record transcripts. The children’s neurodevelopment was repeatedly assessed for five domains (communication, gross motor, fine motor, problem solving, and personal-social), using the Ages and Stages Questionnaires, Third Edition, at six time points from age 6 to 36 months. After adjusting for potential confounders, the associations between exposure to epidural analgesia during labor and children’s neurodevelopment at each time point were assessed.

**Results:**

Of the 42,172 children with valid data at all six time points, 938 (2.4%) were born to mothers who received epidural analgesia during labor. Maternal exposure to epidural analgesia was associated with neurodevelopmental delays during the first 3 years after birth. Delay risks in gross and fine motor domains were the greatest at 18 months (adjusted odds ratio (aOR) [95% confidence interval (CI)]: 1.40 [1.06, 1.84] and 1.54 [1.17, 2.03], respectively), subsequently decreasing. Delay risks in communication and problem-solving domains were significantly high at 6 and 24 months, and remained significant at 36 months (aOR [95% CI]: 1.40 [1.04, 1.90] and 1.28 [1.01, 1.61], respectively). Exposure to epidural analgesia was also associated with the incidence of problem solving and personal-social delays from 18 to 24 months old. Neurodevelopmental delay risks, except for communication, were dominant in children born to mothers aged ≥30 years at delivery.

**Conclusions:**

This study showed that maternal exposure to epidural analgesia during labor was associated with neurodevelopmental delays in children during the first 3 years after birth.

**Supplementary information:**

The online version contains supplementary material available at https://doi.org/10.1265/ehpm.22-00088.

## Introduction

Epidural analgesia is an effective and widely accepted method of providing pain relief during labor [1, 2]. Many studies have reported that epidural analgesia provides better pain relief during labor than systemic opioids or other techniques [3, 4]. The use of epidural analgesia during labor has increased internationally in recent years, and is currently used in 20–70% of all deliveries worldwide [3, 5–7]. However, while the proportion of analgesia administered during labor has increased from 4.6% in 2014 to 6.1% in 2016 [8], the level of use is markedly lower in Japan than in other countries, owing to cultural backgrounds and a shortage of obstetric anesthesia providers [9].

Although some studies have shown adverse effects of epidural analgesia during labor, such as low Apgar scores and admissions to the neonatal intensive care unit [7, 10, 11], others have found no risks associated with epidural analgesia for either mothers or children [1, 3, 12–15]. A systematic review found no relationship between use of epidural analgesia and neonatal morbidity [3]. However, despite the low anesthetics doses administered during epidural analgesia, they cross the placenta and enter the fetus [16, 17]. Additionally, epidural analgesia during labor was also reported to be associated with morphological changes in neonatal brains [18]. Most previous studies have evaluated the effects of epidural analgesia on children only during the peri- or neonatal period. Some recent retrospective cohort studies reported conflicting results on the association of epidural analgesia with an increased risk of autism spectrum disorders (ASD) in children [19–22]. Although these studies evaluated the children with clinical diagnoses of ASD retrospectively, few prospective cohort studies have reported on the long-term neurodevelopment of children born to mothers who received epidural analgesia during labor.

We previously reported that surgical procedures under general anesthesia in infancy were associated with the risk of neurodevelopmental delay at 1 year of age, using data from the Japan Environment and Children’s Study (JECS), an ongoing large-scale, prospective, nationwide birth cohort study in Japan [23]. In the present study, we investigated associations between epidural analgesia exposure during labor and children’s neurodevelopment of during the first 3 years of life.

## Methods

### Study design and participants

Data used in this study were obtained from the JECS, which registered over 100,000 pregnant women between January 2011 and March 2014 in 15 regional centers throughout Japan. The JECS has been designed to investigate mothers and children, to investigate the effects of various environmental factors on the health of the children. This study was registered to the UMIN Clinical Trials Registry (number: UMIN000030786). Details of the protocol and baseline profiles of participants in the JECS have been described elsewhere [24–26]. The JECS protocol was reviewed and approved by the Institutional Review Board on Epidemiological Studies of the Ministry of the Environment, and by the ethics committees of all participating institutions. Written informed consent was obtained from all participants.

After registration, the expecting mothers answered self-administered questionnaires twice during pregnancy: in the first and second/third trimester. The medical records of mothers and children were transcribed by physicians, midwives, nurses, and/or research coordinators immediately after delivery, and 1 month after birth. Children were followed up mainly through self-administered questionnaires completed by their mothers or guardians at 1 month after birth, and thereafter once every 6 months from 6 months to 3 years of age. Each child’s neurodevelopment was assessed using the Japanese translation of the Ages and Stages Questionnaires, Third Edition (ASQ-3) [27, 28], a parent-completed screening tool, at a total of six time points from 6 months to 3 years of age.

**Fig. 1:**
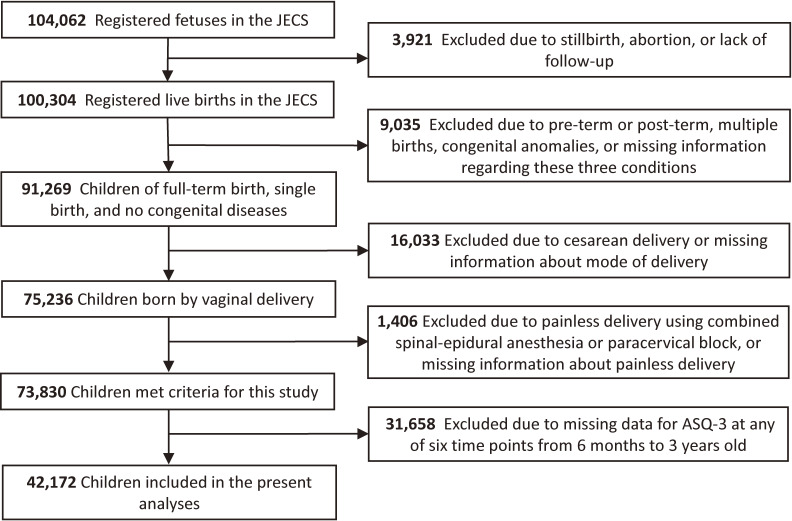
Flowchart showing the process used to select study participants.

Figure 1 shows a flowchart of the inclusion process for this study. Of the 103,060 pregnancies and 104,062 fetuses registered in the JECS, 100,304 were live births. Among these, preterm (<37 weeks of gestation) or post-term (>41 weeks of gestation) births, multiple births, and infants with congenital anomalies, all of which can affect development, were excluded. To investigate the effects of epidural analgesia during labor, as the most common method of painless delivery, we also excluded children delivered by cesarean section, those with missing information about delivery mode or type of painless delivery, and those born by painless delivery using combined spinal-epidural anesthesia or a paracervical block. Furthermore, of the 73,830 children who met the criteria for this study, children without valid data from the ASQ-3 at any of the six time points from 6 months to 3 years were excluded to investigate the progress of neurodevelopment among the same children. Finally, a total of 42,172 children were included in the present analyses. This study is based on the “jecs-ta-20190930” dataset, released in October 2019 and revised in April 2020.

### Exposure and outcomes

In this study, epidural analgesia administered during labor and delivery was used as the exposure variable. Information on delivery mode and whether mothers experienced a painless delivery were obtained from medical record transcripts just after delivery. When painless delivery was performed, the type of anesthesia used (epidural anesthesia, combined spinal-epidural anesthesia, or paracervical block) was confirmed.

Neurodevelopmental delays in the child were assessed using the ASQ-3, a parent-completed developmental screening tool designed for children from 1 to 66 months of age [29–31]. The ASQ-3 comprises 30 questions across five developmental domains (communication, gross motor, fine motor, problem-solving, and personal-social). For each question, the parent answers “yes,” “sometimes,” or “not yet.” These answers are allocated scores of 10, 5, or 0 points, respectively, and the sum of individual scores is calculated. If the score for each domain is less than the cut-off level, the child is referred for further assessment. Details of the ASQ-3 were provided by Squires et al [27]. The present study used the Japanese translation of the ASQ-3 (J-ASQ-3), which has been validated using adjusted cut-off scores for Japanese children [28]. When the score for each domain was below the Japanese cut-off, the child was considered to have a neurodevelopmental delay in that domain.

### Covariates

Covariates were chosen based on findings derived from previous studies into the effects of epidural analgesia during labor [6–10]. Data on covariates were obtained from responses to the self-administered questionnaires and medical record transcripts. Maternal characteristics included maternal age at delivery, parity, delivery mode, duration of labor, comorbidity (diabetes and hypertension), body mass index before pregnancy, occupation in the first trimester, smoking status, alcohol consumption, educational level, annual household income, and marital status at 6 months after childbirth. In addition, the children’s characteristics included sex, birth weight, Apgar score at 5 min after birth, feeding methods in infancy, nursery attendance at 6 and 12 months, screen time at 12 months, and sibling cohabitations [25, 32]. The study area was included as a covariate to consider geographical variations in the use of epidural analgesia during labor.

### Statistical analysis

The characteristics of mothers and children were compared between the groups exposed and unexposed to epidural analgesia during labor. For understanding children’s neurodevelopment, scores in each J-ASQ-3 domain at each time point were first compared between the exposed and unexposed groups. Next, children below and above the cut-off score for each domain at each time point were termed “delayed” and “normal,” respectively, and proportions were compared between groups. Subsequently, multiple logistic regression analyses were performed to estimate associations between epidural analgesia during labor and neurodevelopment, after adjusting for the above covariates. Results were shown as adjusted odds ratios (aORs) and 95% confidence intervals (CIs) of risk of neurodevelopmental delay in the group exposed to epidural analgesia. Additionally, associations between epidural analgesia and the incidence of neurodevelopmental delay at every 6 months after 18 months old were estimated. In these analyses, we examined neurodevelopmental delays after 18 months old, because the ASQ-3 may not be accurate for children under 13 months old [33]. Neurodevelopmental delay at 24 months onset in children without delay at 18 months indicated delay onset during 18–24 months. Similarly, neurodevelopmental delay onset at 30 and 36 months was assessed in children without delay at 24 and 30 months, respectively. Moreover, we examined the relationship between epidural analgesia and neurodevelopmental delays after stratifying by maternal age at delivery (<30 years/≥30 years), because the epidural analgesia administration was higher among older mothers; older maternal age was associated with decreased ASQ-3 scores [34]. Furthermore, we also performed stratified analyses by parity to address differences in the effects of epidural analgesia between primiparas and multiparas.

We applied a multiple imputation method for missing variables to reduce potential non-response bias from missing data and to improve the precision of estimates. The 20 datasets for each imputed variable were created and merged to estimate aORs and 95% CIs [35, 36]. For sensitivity analyses, we also estimated associations between epidural analgesia and neurodevelopment without a multiple imputation method. All statistical analyses were performed using SPSS Statistics version 27 (IBM Corp., Armonk, NY).

## Results

### Characteristics of participants and scores for each J-ASQ-3 domain

The characteristics of mothers and children are shown in Table 1. Of the 42,172 children included in this study, 938 (2.2%) were born to mothers who received epidural analgesia during labor. This percentage was similar to the 2.1% among those excluded from this analysis because of missing J-ASQ-3 scores from 6 to 36 months old (Table S1). Mothers who received epidural analgesia were associated with higher frequencies of induced delivery, vacuum or forceps delivery, and longer duration of labor. Regarding the characteristics of children, the proportion of children living with siblings was lower in the group exposed to epidural analgesia than in the unexposed group. The children’s sex and the proportion of 5-min Apgar scores <7 did not differ between the groups. The characteristics of the children included in this analysis were similar to those of the excluded children (Table S1).

**Table 1:** Characteristics of study participants

**Characteristics**	**Unexposed to epidural analgesia during labor** **(n = 41,234)**		**Exposed to epidural analgesia during labor** **(n = 938)**	
Maternal characteristics				
Age at delivery, years				
Mean (SD)	31.3	(4.8)	32.5	(4.7)
<20	420	(1.0)	5	(0.5)
20–29	14,705	(35.7)	244	(26.0)
30–39	24,453	(59.3)	623	(66.4)
≥40	1,655	(4.0)	66	(7.0)
Parity				
Primipara	17,419	(43.3)	495	(53.4)
Multipara	22,830	(56.7)	432	(46.6)
Delivery mode				
Spontaneous	29,410	(71.3)	239	(25.5)
Induced	8,809	(21.4)	497	(53.0)
Vacuum or forceps	3,015	(7.3)	202	(21.5)
Duration of labor, hours				
Mean (SD)	8.7	(7.3)	10.9	(9.6)
<4.0	10,079	(25.2)	158	(17.3)
4.0–7.9	15,649	(39.2)	437	(47.8)
≥8.0	14,240	(35.6)	319	(34.9)
Comorbidity				
Diabetes	348	(0.8)	6	(0.6)
Hypertension	292	(0.7)	9	(1.0)
BMI before pregnancy				
Mean (SD)	20.9	(2.9)	20.8	(2.7)
<18.5	6,992	(17.0)	177	(18.9)
18.5–24.9	30,894	(75.0)	697	(74.4)
≥25	3,326	(8.1)	63	(6.7)
Occupation in the first trimester				
Yes	26,660	(66.3)	581	(64.8)
No	13,526	(33.7)	316	(35.2)
Smoking status				
Never-smoked	25,701	(62.3)	545	(58.1)
Ex-smoker (quit before pregnancy)	9,524	(23.1)	248	(26.4)
Ex-smoker (quit during early pregnancy)	4,453	(10.8)	84	(9.0)
Current smoker	1,223	(3.0)	31	(3.3)
Alcohol consumption				
Never drank	14,569	(35.5)	305	(33.6)
Ex-drinker	22,317	(54.5)	521	(57.3)
Current drinker	4,099	(10.0)	83	(9.1)
Education, years				
<13	13,257	(32.3)	247	(26.4)
13–15	17,688	(43.1)	394	(42.1)
≥16	10,057	(24.5)	295	(31.5)
Annual household income				
<4,000,000 JPY	14,777	(38.2)	246	(28.0)
4,00,000–7,999,999 JPY	19,547	(50.5)	468	(53.2)
≥8,000,000 JPY	4,358	(11.3)	166	(18.9)
Marital status at 6 months after childbirth				
Married (including common-law marriage)	40,441	(98.1)	924	(98.5)
Divorced	209	(0.5)	6	(0.6)
Widowed	14	(0.0)	1	(0.1)
Others	308	(0.7)	3	(0.3)

Child’s characteristics				
Sex				
Male	21,023	(51.0)	461	(49.1)
Female	20,211	(49.0)	477	(50.9)
Birth weight				
Mean (SD)	3080.7	(356.4)	3048.2	(351.6)
<2500	1,820	(4.4)	55	(5.9)
2500–3999	39,051	(94.7)	879	(93.8)
≥4000	357	(0.9)	3	(0.3)
Apgar score at 5 min				
<7	117	(0.3)	0	(0.0)
≥7	39,742	(99.7)	920	(100.0)
Feeding methods in infancy				
Breast	20,335	(49.3)	347	(37.0)
Bottle	3,260	(7.9)	112	(11.9)
Mixed	17,639	(42.8)	479	(51.1)
Nursery attendance at 6 months old				
Yes	2,350	(5.7)	54	(5.8)
No	38,831	(94.3)	883	(94.2)
Nursery attendance at 12 months old				
Yes	9,983	(24.3)	219	(23.4)
No	31,129	(75.7)	716	(76.6)
Screen time at 12 months old				
No viewing	4,250	(10.4)	107	(11.5)
<1	13,926	(34.1)	302	(32.5)
1–<2	12,186	(29.8)	285	(30.6)
2–<4	7,950	(19.4)	171	(18.4)
≥4	2,565	(6.3)	65	(7.0)
Sibling cohabitations				
No	19,451	(47.3)	511	(56.0)
Yes	21,671	(52.7)	402	(44.0)

Data represent mean (SD) or n (%).

Abbreviations: SD, standard deviation; BMI, body mass index; JPY, Japanese yen.

The mean scores for each J-ASQ-3 domain at each time are shown in Table S2. All scores were lower in the group exposed to epidural analgesia than in the unexposed group, and most of the differences were significant (*p* < .05 for ≥4 time points for all 5 domains). The numbers and proportions of children below the cut-off score for each J-ASQ-3 domain at each time point are shown in Table 2. The proportions of children with these scores were higher in the exposed group than in the unexposed group, except scores for the personal-social domain at 6 months. These proportions were broadly similar between children included and excluded in this analysis (Table S3).

**Table 2:** Neurodevelopmental delay in each domain of the J-ASQ-3 among children, with or without epidural analgesia

**Domain**	**Cut-off scores for each item^a^**	**Unexposed to epidural analgesia during labor** **(n = 41,234)**		**Exposed to epidural analgesia during labor** **(n = 938)**	
	
		**n**	**(%)**	**n**	**(%)**
Communication					
6 months	22.93	231	(0.6)	10	(1.1)
12 months	4.53	42	(0.1)	2	(0.2)
18 months	5.82	831	(2.0)	29	(3.1)
24 months	14.33	1,474	(3.6)	50	(5.3)
30 months	26.01	1,844	(4.5)	49	(5.2)
36 months	29.95	1,498	(3.6)	50	(5.3)
Gross motor					
6 months	15.12	4,091	(9.9)	112	(11.9)
12 months	9.43	2,156	(5.2)	59	(6.3)
18 months	37.59	1,702	(4.1)	61	(6.5)
24 months	39.13	2,174	(5.3)	72	(7.7)
30 months	38.36	1,574	(3.8)	46	(4.9)
36 months	39.26	1,636	(4.0)	42	(4.5)
Fine motor					
6 months	16.24	2,021	(4.9)	49	(5.2)
12 months	25.47	2,187	(5.3)	54	(5.8)
18 months	26.76	1,663	(4.0)	61	(6.5)
24 months	33.48	782	(1.9)	25	(2.7)
30 months	21.03	2,275	(5.5)	65	(6.9)
36 months	27.91	2,910	(7.1)	77	(8.2)
Problem solving					
6 months	26.27	4,281	(10.4)	123	(13.1)
12 months	15.37	2,039	(4.9)	55	(5.9)
18 months	15.93	1,601	(3.9)	48	(5.1)
24 months	29.38	1,585	(3.8)	55	(5.9)
30 months	25.78	2,177	(5.3)	65	(6.9)
36 months	30.03	2,851	(6.9)	85	(9.1)
Personal-social					
6 months	22.53	1,424	(3.5)	32	(3.4)
12 months	20.93	447	(1.1)	17	(1.8)
18 months	34.87	943	(2.3)	33	(3.5)
24 months	34.30	1,017	(2.5)	46	(4.9)
30 months	39.95	1,275	(3.1)	44	(4.7)
36 months	40.27	1,219	(3.0)	37	(3.9)

^a^Cut-off scores for Japanese children [28] were used.

Abbreviations: J-ASQ-3, the Japanese translation of the Ages and Stages Questionnaires, Third Edition.

### Association between epidural analgesia during labor and the prevalence of neurodevelopmental delay

**Fig. 2:**
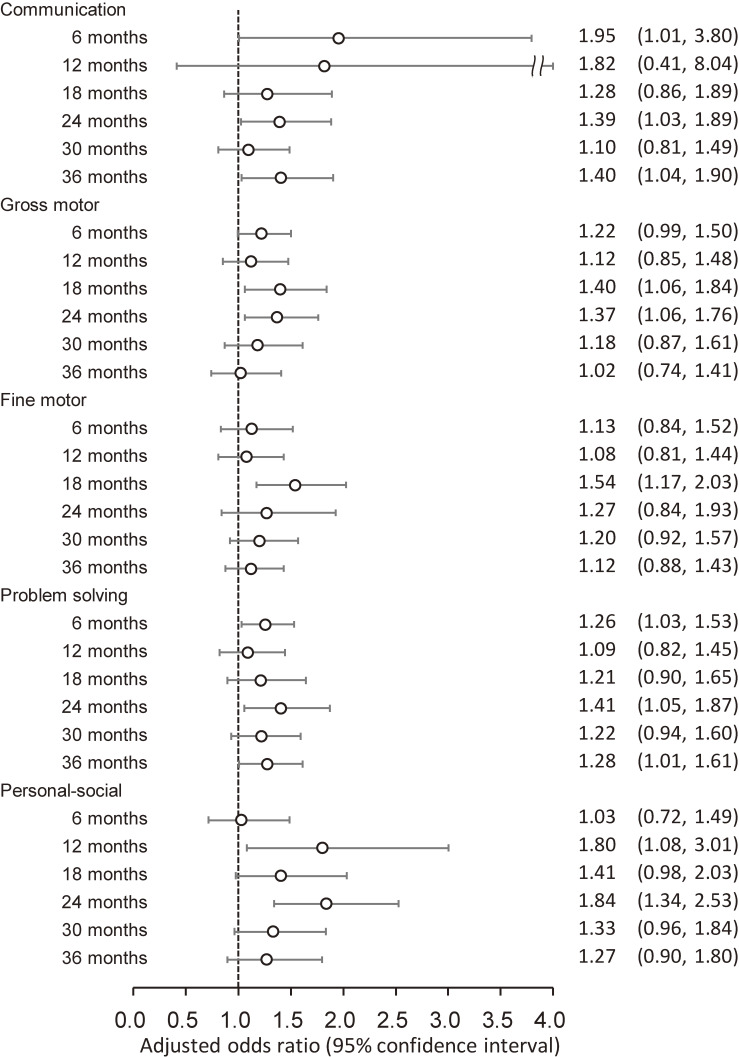
Association of epidural analgesia during labor with neurodevelopmental delay among children Adjusted odds ratios (95% confidence intervals) for neurodevelopmental delay in each domain of the J-ASQ-3 among children born to mothers who received epidural analgesia during labor are shown from age 6 to 36 months. Adjusted for maternal age at delivery, parity, delivery mode, duration of labor, comorbidity (diabetes and hypertension), body mass index before pregnancy, occupation in the first trimester, smoking status, alcohol consumption, educational level, annual household income, marital status at 6 months after childbirth, child’s sex, birth weight, Apgar score at 5 min after birth, feeding methods in infancy, nursery attendance, screen time at 12 months old, siblings cohabiting with the child, and study area. A multiple imputation method was used to reduce potential selection bias from missing variables. Abbreviations: J-ASQ-3, the Japanese translation of the Ages and Stages Questionnaires, Third Edition.

The results of logistic regression analyses for associations between epidural analgesia during labor and each J-ASQ-3 domain at each time point are shown in Fig. 2. For communication, children born to mothers who received epidural analgesia during labor displayed significantly increased risks of neurodevelopmental delays at 6, 24, and 36 months old (aOR [95%CI]: 1.95 [1.01, 3.80], 1.39 [1.03, 1.89], and 1.40 [1.04, 1.90], respectively). Concerning gross and fine motor development, neurodevelopmental delay risks were significantly higher at 18 months (aOR [95%CI]: 1.40 [1.06, 1.84] and 1.54 [1.17, 2.03], respectively). Thereafter, aORs in both domains gradually decreased; however, gross motor delay risk remained significantly high at 24 months (aOR [95%CI]: 1.37 [1.06, 1.76]). Neurodevelopmental delay risks in problem solving were significantly higher at 6, 24, and 36 months, although no clear trend was apparent during the first 3 years. Personal-social delay risks were significantly high at 12 and 24 months.

**Fig. 3:**
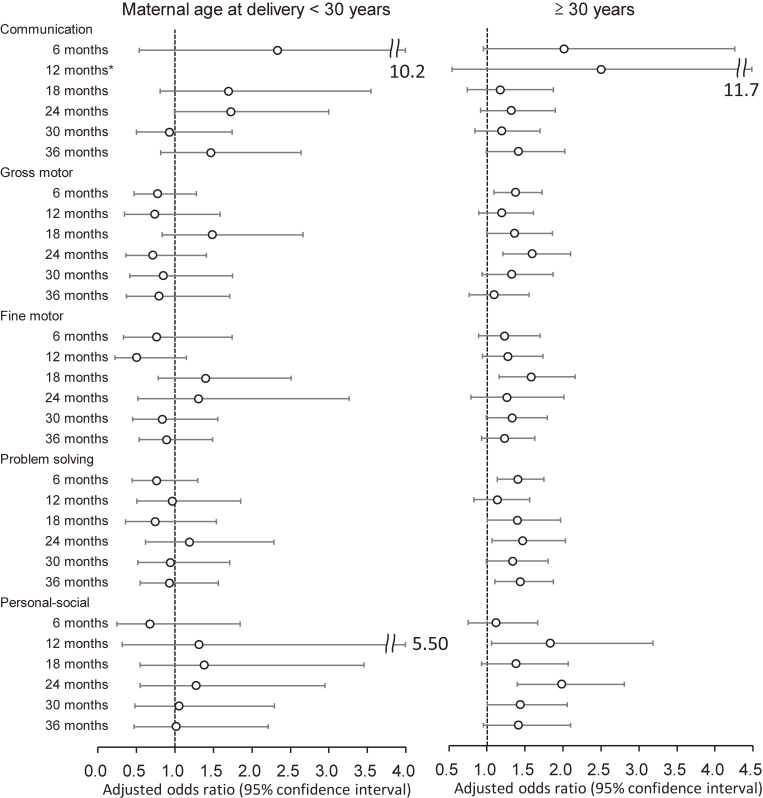
Association of epidural analgesia with neurodevelopmental delay among children, by maternal age Adjusted odds ratios (95% confidence intervals) for neurodevelopmental delay in each domain of the J-ASQ-3 among children born to mothers who received epidural analgesia during labor are shown from age 6 to 36 months, stratified by maternal age at delivery (<30 years/≥30 years). Adjusted for parity, delivery mode, duration of labor, comorbidity (diabetes and hypertension), body mass index before pregnancy, occupation in the first trimester, smoking status, alcohol consumption, educational level, annual household income, marital status at 6 months after childbirth, child’s sex, birth weight, Apgar score at 5 min after birth, feeding methods in infancy, nursery attendance, screen time at 12 months old, siblings cohabiting with the child, and study area. A multiple imputation method was used to reduce potential selection bias from missing variables. For communication delay at 12 months in <30 years, adjusted odds ratio could not be calculated due to the small number of the subjects. Abbreviations: J-ASQ-3, the Japanese translation of the Ages and Stages Questionnaires, Third Edition.

The results were similar in sensitivity analyses without the multiple imputation method (Table S4). In stratified analyses by maternal age at delivery, the associations between epidural analgesia during labor and neurodevelopmental delay, except communication delay, were dominant in children born to mothers aged ≥30 years (Fig. 3). Conversely, stratified analyses by parity revealed that personal-social delay risks were similar in primiparas and multiparas, and other domains’ delay risks were larger in multiparas (Table S5).

### Association between epidural analgesia during labor and the incidence of neurodevelopmental delay

**Fig. 4:**
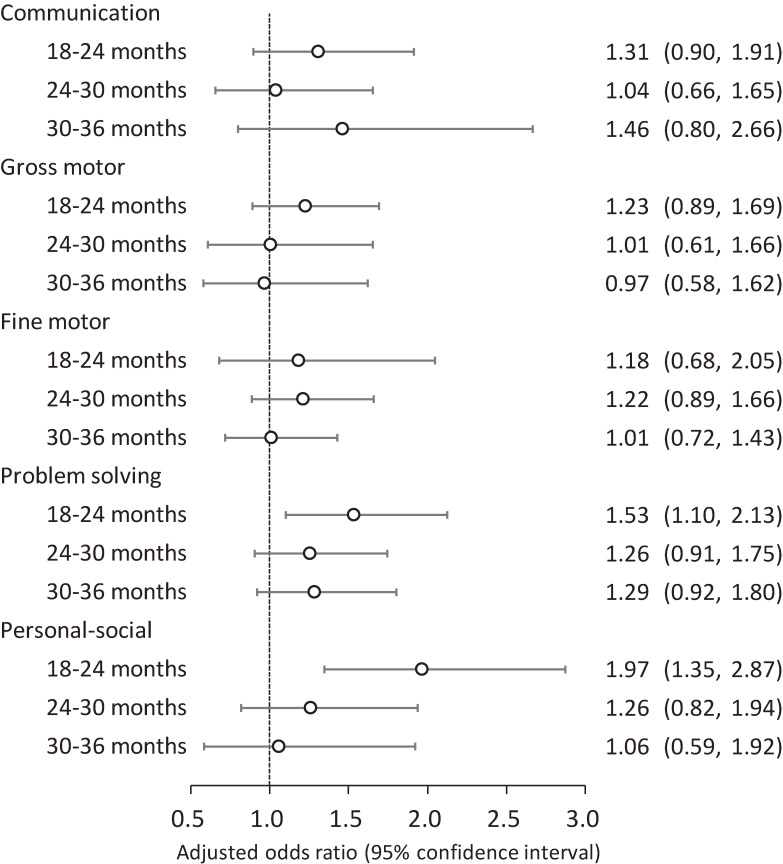
Association of epidural analgesia during labor with the incidence of neurodevelopmental delay among children Adjusted odds ratios (95% confidence intervals) for the incidence of neurodevelopmental delay in each domain of the J-ASQ-3 among children born to mothers who received epidural analgesia during labor are shown, every 6 months after 18 months. Adjusted for maternal age at delivery, parity, delivery mode, duration of labor, comorbidity (diabetes and hypertension), body mass index before pregnancy, occupation in the first trimester, smoking status, alcohol consumption, educational level, annual household income, marital status at 6 months after childbirth, child’s sex, birth weight, Apgar score at 5 min after birth, feeding methods in infancy, nursery attendance, screen time at 12 months old, siblings cohabiting with the child, and study area. A multiple imputation method was used to reduce potential selection bias from missing variables. Abbreviations: J-ASQ-3, the Japanese translation of the Ages and Stages Questionnaires, Third Edition.

**Fig. 5:**
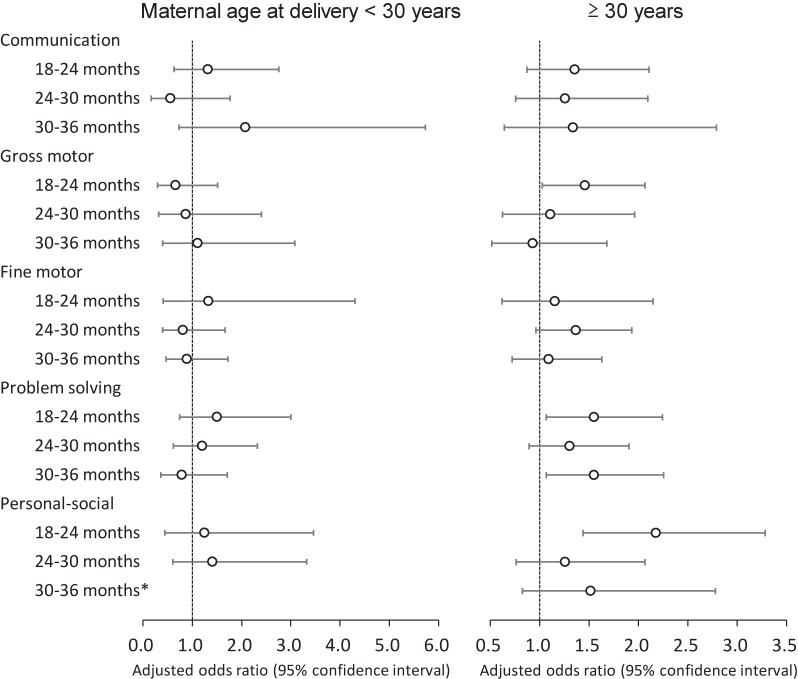
Association of epidural analgesia with the incidence of neurodevelopmental delay among children, by maternal age Adjusted odds ratios (95% confidence intervals) for the incidence of neurodevelopmental delay in each domain of the J-ASQ-3 among children born to mothers who received epidural analgesia during labor are shown, every 6 months after 18 months, stratified by maternal age at delivery (<30 years/≥30 years). Adjusted for parity, delivery mode, duration of labor, comorbidity (diabetes and hypertension), body mass index before pregnancy, occupation in the first trimester, smoking status, alcohol consumption, educational level, annual household income, marital status at 6 months after childbirth, child’s sex, birth weight, Apgar score at 5 min after birth, feeding methods in infancy, nursery attendance, screen time at 12 months old, siblings cohabiting with the child, and study area. A multiple imputation method was used to reduce potential selection bias from missing variables. For personal-social delay during 30–36 months in <30 years, adjusted odds ratio could not be calculated due to the small number of the subjects. Abbreviations: J-ASQ-3, the Japanese translation of the Ages and Stages Questionnaires, Third Edition.

The results of logistic regression analyses for associations between epidural analgesia during labor and the incidence of neurodevelopmental delay are shown in Fig. 4. Risks of the incidence of problem solving and personal-social delays were significantly high from 18 to 24 months old (aOR [95%CI]: 1.53 [1.10, 2.13] and 1.97 [1.35, 2.87], respectively). The incidence of neurodevelopmental delay from 24 to 36 months old was not significant in any domain. Analyses without the multiple imputation method showed similar results (Table S6). In stratified analyses by maternal age at delivery, the incidence of gross motor, problem solving and personal-social delay risks was significantly high from age 18 to 24 months in children born to mothers aged ≥30 years. Delay onset during 30–36 months for problem solving was also significant. In contrast, no significant association was observed in children born to mothers aged <30 years (Fig. 5). Conversely, in stratified analyses by parity, associations between epidural analgesia during labor and incidence of personal-social delay were strong in primiparas; however, associations with gross and fine motor and problem-solving delays were strong in multiparas (Table S7).

## Discussion

This study investigated the progress of neurodevelopment for children born to mothers who received epidural analgesia during labor. Maternal exposure to epidural analgesia was associated with neurodevelopmental delays in five domains of the J-ASQ-3 during the first 3 years after birth. The findings suggested that epidural analgesia during labor may affect the neurodevelopment of children and that the effects may persist up to 36 months old. This is the first study to report the longitudinal characteristics of neurodevelopment in children born to mothers who received epidural analgesia during labor.

Associations between exposure to epidural analgesia during labor and neurodevelopmental outcomes in children have been investigated, mostly in retrospective studies. While many studies have found no adverse effects of epidural analgesia on children [1, 3, 12–15], other studies have reported associations between exposure to epidural analgesia and outcomes in children [7, 10, 11]. However, most previous studies have investigated only peri- and neonatal outcomes among children born to mothers who received epidural analgesia during labor, and findings about the potential long-term effects of epidural analgesia on neurodevelopment in childhood have been limited [3, 37]. One animal study reported that epidural analgesia during labor altered the normal course of behavioral development in rhesus monkeys [38]. Conversely, a human study found that the use of neuraxial analgesia during labor was not associated with learning disabilities in childhood [39]. In a retrospective birth cohort study, Qiu et al. [21] reported that maternal epidural analgesia during labor increased the risk of ASD in children. Recently, Hanley et al. [19] has also reported that epidural analgesia was associated with a small increased risk of ASD; however, two other studies found no association between epidural analgesia and ASD incidence in children [20, 21]. Thus, consensus remains lacking on long-term neurodevelopment in children born to mothers who received epidural analgesia during labor, and study designs, outcomes analyzed, and follow-up periods have differed markedly between studies.

We found neurodevelopmental delays during the first 3 years after birth in children exposed to epidural analgesia during labor. The mechanisms through which epidural analgesia increases neurodevelopmental delay risk remain unknown. Although epidural analgesia is known to prolong the duration of labor [40], prolonged labor has not been demonstrated to be associated with increased risk of ASD [41–43]. Qiu et al. [21] therefore suggested that prolonged exposure to anesthesia may be a risk factor for ASD. In the present study, there was no significant association between duration of epidural analgesia and children’s neurodevelopment (data not shown). However, stratified analyses by parity, which is considered to result in differences in the progress of labor, revealed that personal-social delay and other delays associated with epidural analgesia were prominent among primiparas and multiparas, respectively.

In this study, the progress of neurodevelopmental delays in children exposed to epidural analgesia during delivery differed among the five domains of J-ASQ-3. The number of children with communication delay was very small at 6 and 12 months old; this might have resulted from the considerably lower cut-off score for the domain at each time point for Japanese children than the original score [23, 28]. In addition, Yue et al. [33] pointed out that the ASQ-3 may not be accurate for children aged below 13 months. The delay risks in gross and fine motor domains were the greatest at 18 months, but decreased to become insignificant with the growth of the child. In contrast, the delay risks in communication and problem-solving domains remained significantly high at 36 months, and a significant delay risk in the personal-social domain persisted from 12 to 30 months of age. These results suggest that the effects on delays in these domains may persist until the children are at least 3 years old. The incidence of neurodevelopmental delay risks from age 18 to 24 months were also significant for the problem-solving and personal-social domains. The ASQ-3 is used as a screening tool to detect neurodevelopmental disorders in children [29–31]. Delays in the communication domain have been reported to indicate an initial concern for ASD [44]. Delays in the problem-solving and personal-social domains are also likely to be associated with developmental disorders.

Increased maternal age has been reported to be associated with decreased gross motor and personal-social scores [34]. Conversely, epidural analgesia administration was higher among older mothers in this study. In stratified analyses by maternal age at delivery, the association between epidural analgesia and neurodevelopmental delays was dominant in children born to mothers aged ≥30 years. In addition, the incidence of gross motor, problem solving, and personal-social delay risks from age 18 to 24 months was observed in children born to mothers aged ≥30 years, consistent to the previous report [34].

The main strength of this study was that we were able to examine associations between the neurodevelopment of children and exposure to epidural analgesia during labor, based on a large-scale birth cohort study. In addition, neurodevelopmental outcomes were repeatedly assessed six times from 6 to 36 months old, and long-term effects of exposure to epidural analgesia on neurodevelopment in children were evaluated. Furthermore, we were able to adjust for many confounding factors, because various information from mothers and children during the pre-, peri-, and postnatal periods was obtained in this study.

On the other hand, several limitations must be considered when interpreting the findings of the present study. First, details of epidural analgesia, including the kind and dosage of anesthetic drug used, were unknown. Moreover, unadjusted potential confounding factors may have been involved in the association between epidural analgesia and neurodevelopmental delay. For example, several paternal factors (e.g., genetic predisposition, viral or bacterial infection) may need to be considered as potential confounders. Second, we could not evaluate the effects of maternal fever during delivery related to epidural analgesia. The incidence of maternal fever has been reported to be high among women who received epidural analgesia [45], and could be associated with neurodevelopment in children [46, 47]. Third, many children of participants in the JECS study were excluded from analyses, because the subjects in this study were limited to children with valid data from all six time points. Additionally, the result at each time point was analyzed independently, giving rise to multiple tests. However, we believe that the effects of epidural analgesia during labor on the progress of neurodevelopment among the same children were accurately evaluated. Furthermore, the characteristics and proportions of children below the cut-off score were similar between those included and excluded in this analysis (Tables S1 and S3). Fourth, neurodevelopmental delays were assessed using the J-ASQ-3, a parent-completed screening tool, and not considering the doctor’s diagnosis. Mothers who received epidural analgesia may be more aware of their child’s neurodevelopmental delay. However, the ASQ-3 is widely used to assess neurodevelopmental delays in children, and is appropriate for a large-scale epidemiological study. Finally, neurodevelopmental outcomes were evaluated during the first 3 years after birth. Considerable individual neurodevelopmental variations are present in early childhood, and whether the delays observed in this study can predict future prognosis is unclear. The present study intends to follow up children, and the longer-term effects of epidural analgesia during labor on neurodevelopmental disorders should be further elucidated.

## Conclusions

Our findings indicated that delay risks in gross and fine motor domains were the greatest at 18 months in children born to mothers who received epidural analgesia during labor, and thereafter gradually decreased. In contrast, delay risks in communication and problem-solving domains were significantly high at 6 and 24 months and remained significant at 36 months of age. Maternal exposure to epidural analgesia was also associated with the incidence of problem solving and personal-social delays from age 18 to 24 months. Neurodevelopmental delay risks, except for communication, were dominant in children born to mothers aged ≥30 years at delivery. These findings suggest that epidural analgesia during labor affects the neurodevelopment of children and that the effects may persist up to 36 months of age.

## Abbreviations

aOR: adjusted odds ratio
ASD: autism spectrum disorders
ASQ-3: Ages and Stages Questionnaires, Third Edition
CI: confidence interval
J-ASQ-3: the Japanese translation of the Ages and Stages Questionnaires, Third Edition
JECS: Japan Environment and Children’s Study

## Supplementary information

**Additional file 1:** **Table S1.** Comparison of characteristics between participants included in and excluded from this study. **Table S2.** Scores in each domain of the J-ASQ-3 among children from 6 to 36 months old, with or without epidural analgesia. **Table S3.** Comparison of numbers and proportions of neurodevelopmental delay in each domain of the J-ASQ-3 between participants included in and excluded from this study. **Table S4.** Adjusted odds ratios for neurodevelopmental delay in each domain of the J-ASQ-3 among children born to mothers who received epidural analgesia during labor, from 6 to 36 months old, without multiple imputation method. **Table S5.** Adjusted odds ratios for neurodevelopmental delay in each domain of the J-ASQ-3 among children born to mothers who received epidural analgesia during labor, from 6 to 36 months old, stratified by parity. **Table S6.** Adjusted odds ratios for the incidence of neurodevelopmental delay in each domain of the J-ASQ-3 among children born to mothers who received epidural analgesia during labor, every 6 months after 18 months old, without multiple imputation method. **Table S7.** Adjusted odds ratios for the incidence of neurodevelopmental delay in each domain of the J-ASQ-3 among children born to mothers who received epidural analgesia during labor, every 6 months after 18 months old, stratified by parity.

## Declarations

### Availability of data and materials

The JECS data are not publicly available due to ethical restrictions and the legal framework of Japan. All inquiries about access to the data should be sent to the JECS Programme Office, National Institute for Environmental Studies (jecs-en@nies.go.jp).
