# Supplementary material for: Association of epidural analgesia during labor with neurodevelopment of children during the first three years: the Japan Environment and Children’s Study
Source: Environ Health Prev Med. 2022 Sep 28;27:37. doi: 10.1265/ehpm.22-00088 (PMC9556973; doi:10.1265/ehpm.22-00088)
Supplement: Supplementary file 1 — Additional file 1: Table S1. Comparison of characteristics between participants included in and excluded from this study. Table S2. Scores in each domain of the J-ASQ-3 among children from 6 to 36 months old, with or without epidural analgesia. Table S3. Comparison of numbers and proportions of neurodevelopmental delay in each domain of the J-ASQ-3 between participants included in and excluded from this study. Table S4. Adjusted odds ratios for neurodevelopmental delay in each domain of the J-ASQ-3 among children born to mothers who received epidural analgesia during labor, from 6 to 36 months old, without multiple imputation method. Table S5. Adjusted odds ratios for neurodevelopmental delay in each domain of the J-ASQ-3 among children born to mothers who received epidural analgesia during labor, from 6 to 36 months old, stratified by parity. Table S6. Adjusted odds ratios for the incidence of neurodevelopmental delay in each domain of the J-ASQ-3 among children born to mothers who received epidural analgesia during labor, every 6 months after 18 months old, without multiple imputation method. Table S7. Adjusted odds ratios for the incidence of neurodevelopmental delay in each domain of the J-ASQ-3 among children born to mothers who received epidural analgesia during labor, every 6 months after 18 months old, stratified by parity. [file ehpm-27-037-s001.docx]

**Additional File**

Association of epidural analgesia during labor with neurodevelopment of children during the first three years: the Japan Environment and Children’s Study

Masayuki Shima, Narumi Tokuda, Hideki Hasunuma, Yoshiko Kobayashi, Hiroyuki Tanaka, Hideaki Sawai, Hiroaki Shibahara, Yasuhiro Takeshima, Munetaka Hirose, and the Japan Environment and Children’s Study (JECS) Group

**Table of Contents**

**Table S1.** Comparison of characteristics between participants included in and excluded from this study

**Table S2.** Scores in each domain of the J-ASQ-3 among children from 6 to 36 months old, with or without epidural analgesia

**Table S3.** Comparison of numbers and proportions of neurodevelopmental delay in each domain of the J-ASQ-3 between participants included in and excluded from this study

**Table S4.** Adjusted odds ratios for neurodevelopmental delay in each domain of the J-ASQ-3 among children born to mothers who received epidural analgesia during labor, from 6 to 36 months old, without multiple imputation method

**Table S5.** Adjusted odds ratios for neurodevelopmental delay in each domain of the J-ASQ-3 among children born to mothers who received epidural analgesia during labor, from 6 to 36 months old, stratified by parity

**Table S6.** Adjusted odds ratios for the incidence of neurodevelopmental delay in each domain of the J-ASQ-3 among children born to mothers who received epidural analgesia during labor, every 6 months after 18 months old, without multiple imputation method

**Table S7.** Adjusted odds ratios for the incidence of neurodevelopmental delay in each domain of the J-ASQ-3 among children born to mothers who received epidural analgesia during labor, every 6 months after 18 months old, stratified by parity

**Table S1.** Comparison of characteristics between participants included in and excluded from this study

| Characteristics | | Included in this analysis  (n = 42,172) | | Excluded from this analysis  (n = 31,658) | |
| --- | --- | --- | --- | --- | --- |
| Epidural analgesia during labor | |  |  |  |  |
|  | Yes | 938 | (2.2) | 665 | (2.1) |
|  | No | 41,234 | (97.8) | 30,993 | (97.9) |
| Maternal characteristics | |  |  |  |  |
| Age at delivery, years | |  |  |  |  |
|  | Mean (SD) | 31.3 | (4.8) | 30.2 | (5.2) |
|  | <20 | 425 | (1.0) | 981 | (3.1) |
|  | 20–29 | 14,949 | (35.4) | 13,141 | (41.5) |
|  | 30–39 | 25,076 | (59.5) | 16,508 | (52.2) |
|  | ≥40 | 1,721 | (4.1) | 1,023 | (3.2) |
| Parity | |  |  |  |  |
|  | Primipara | 17,914 | (43.5) | 10,932 | (35.3) |
|  | Multipara | 23,262 | (56.5) | 20,002 | (64.7) |
| Delivery mode | |  |  |  |  |
|  | Spontaneous | 29,649 | (70.3) | 22,725 | (71.8) |
|  | Induced | 9,306 | (22.1) | 6,855 | (21.7) |
|  | Vacuum or forceps | 3,217 | (7.6) | 2,078 | (6.6) |
| Duration of labor, hours | |  |  |  |  |
|  | Mean (SD) | 8.7 | (7.4) | 8.2 | (7.0) |
|  | <4.0 | 10,399 | (25.3) | 8,314 | (26.9) |
|  | 4.0–7.9 | 14,596 | (35.5) | 11,343 | (36.7) |
|  | ≥8.0 | 16,149 | (39.2) | 11,281 | (36.5) |
| Comorbidity | |  |  |  |  |
|  | Diabetes | 354 | (0.8) | 270 | (0.9) |
|  | Hypertension | 301 | (0.7) | 265 | (0.8) |
| BMI before pregnancy | |  |  |  |  |
|  | Mean (SD) | 20.9 | (2.9) | 21.2 | (3.3) |
|  | <18.5 | 7,169 | (17.0) | 5,232 | (16.6) |
|  | 18.5–24.9 | 31,591 | (75.0) | 22,937 | (72.6) |
|  | ≥25 | 3,389 | (8.0) | 3,434 | (10.9) |
| Occupation in the first trimester | |  |  |  |  |
|  | Yes | 27,241 | (66.3) | 20,018 | (67.1) |
|  | No | 13,842 | (33.7) | 9,808 | (32.9) |
| Smoking status | |  |  |  |  |
|  | Never-smoker | 26,246 | (62.8) | 16,104 | (52.6) |
|  | Ex-smoker (quit before pregnancy) | 9,772 | (23.4) | 7,281 | (23.8) |
|  | Ex-smoker (quit during early pregnancy) | 4,537 | (10.9) | 5,087 | (16.6) |
|  | Current smoker | 1,254 | (3.0) | 2,117 | (6.9) |
| Alcohol consumption | |  |  |  |  |
|  | Never-drinker | 14,874 | (35.5) | 10,349 | (33.7) |
|  | Ex-drinker | 22,838 | (54.5) | 17,354 | (56.5) |
|  | Current drinker | 4,182 | (10.0) | 3,022 | (9.8) |
| Education, years | |  |  |  |  |
|  | <13 | 13,504 | (32.2) | 12,904 | (42.4) |
|  | 13–15 | 18,082 | (43.1) | 12,202 | (40.1) |
|  | ≥16 | 10,352 | (24.7) | 5,355 | (17.6) |
| Annual household income | |  |  |  |  |
|  | <4,000,000 JPY | 15,023 | (38.0) | 12,286 | (43.9) |
|  | 4,00,000–7,999,999 JPY | 20,015 | (50.6) | 12,997 | (46.5) |
|  | ≥8,000,000 JPY | 4,524 | (11.4) | 2,685 | (9.6) |
| Marital status at 6 months after childbirth | |  |  |  |  |
|  | Married (including common-law marriage) | 41,365 | (98.7) | 26,000 | (97.3) |
|  | Divorced | 215 | (0.5) | 342 | (1.3) |
|  | Widowed | 15 | (0.0) | 13 | (0.0) |
|  | Others | 311 | (0.7) | 359 | (1.3) |
| Child’s characteristics | |  |  |  |  |
| Sex | |  |  |  |  |
|  | Male | 21,484 | (50.9) | 16,076 | (50.8) |
|  | Female | 20,688 | (49.1) | 15,580 | (49.2) |
| Birth weight | |  |  |  |  |
|  | Mean (SD) | 3,080.0 | (356.3) | 3,089.6 | (362.2) |
|  | <2500 | 1,875 | (4.4) | 1,395 | (4.4) |
|  | 2500–3999 | 39,930 | (94.7) | 29,967 | (94.7) |
|  | ≥4000 | 360 | (0.9) | 290 | (0.9) |
| Apgar score at 5 min | |  |  |  |  |
|  | <7 | 117 | (0.3) | 85 | (0.3) |
|  | ≥7 | 40,662 | (99.7) | 30,360 | (99.7) |
| Feeding methods in infancy | |  |  |  |  |
|  | Breast | 20,682 | (49.0) | 11,604 | (46.8) |
|  | Bottle | 3,372 | (8.0) | 2,807 | (11.3) |
|  | Mixed | 18,118 | (43.0) | 10,370 | (41.8) |
| Nursery attendance at 6 months old | |  |  |  |  |
|  | Yes | 2,404 | (5.7) | 2,478 | (9.2) |
|  | No | 39,714 | (94.3) | 24,448 | (90.8) |
| Nursery attendance at 12 months old | |  |  |  |  |
|  | Yes | 10,202 | (24.3) | 7,879 | (32.0) |
|  | No | 31,845 | (75.7) | 16,734 | (68.0) |
| Screen time at 12 months old | |  |  |  |  |
|  | No viewing | 4,357 | (10.4) | 2,332 | (9.5) |
|  | <1 | 14,228 | (34.0) | 7,979 | (32.6) |
|  | 1–<2 | 12,471 | (29.8) | 7,634 | (31.2) |
|  | 2–<4 | 8,120 | (19.4) | 4,997 | (20.4) |
|  | ≥4 | 2,630 | (6.3) | 1,565 | (6.4) |
| Sibling cohabitations | |  |  |  |  |
|  | No | 19,962 | (47.5) | 12,513 | (40.5) |
|  | Yes | 22,073 | (52.5) | 18,398 | (59.5) |

Data represent mean (SD) or n (%). Percentages were calculated based on the number of valid responses for each item.

Abbreviations: SD, standard deviation; BMI, body mass index, JPY, Japanese yen.

**Table S2.** Scores in each domain of the J-ASQ-3 among children from 6 to 36 months old, with or without epidural analgesia

|  | | Unexposed to epidural analgesia during labor  (n=41,234) | | Exposed to epidural analgesia during labor  (n= 938) | |
| --- | --- | --- | --- | --- | --- |
| Communication | |  |  |  |  |
|  | 6 months | 46.6 | (8.8) | 46.3 | (9.0) |
|  | 12 months | 37.7 | (13.3) | 36.2 | (13.4) |
|  | 18 months | 33.1 | (13.0) | 31.4 | (13.6) |
|  | 24 months | 45.4 | (15.5) | 43.6 | (16.6) |
|  | 30 months | 53.3 | (11.1) | 52.3 | (11.9) |
|  | 36 months | 53.4 | (10.2) | 52.6 | (11.3) |
| Gross motor | |  |  |  |  |
|  | 6 months | 33.6 | (12.6) | 32.7 | (12.7) |
|  | 12 months | 43.3 | (17.2) | 41.9 | (17.6) |
|  | 18 months | 54.9 | (8.9) | 53.6 | (10.2) |
|  | 24 months | 54.1 | (8.6) | 53.2 | (9.6) |
|  | 30 months | 55.0 | (7.7) | 54.4 | (8.7) |
|  | 36 months | 55.7 | (7.8) | 55.1 | (8.4) |
| Fine motor | |  |  |  |  |
|  | 6 months | 40.8 | (13.9) | 40.0 | (13.9) |
|  | 12 months | 48.5 | (11.2) | 48.2 | (11.4) |
|  | 18 months | 50.1 | (10.4) | 49.2 | (11.4) |
|  | 24 months | 50.0 | (7.1) | 49.3 | (7.5) |
|  | 30 months | 47.6 | (12.3) | 46.5 | (13.0) |
|  | 36 months | 49.5 | (12.4) | 49.1 | (13.0) |
| Problem solving | |  |  |  |  |
|  | 6 months | 44.2 | (12.4) | 42.9 | (12.7) |
|  | 12 months | 42.5 | (13.4) | 41.0 | (13.8) |
|  | 18 months | 42.6 | (12.3) | 41.2 | (13.0) |
|  | 24 months | 49.2 | (10.4) | 48.0 | (11.6) |
|  | 30 months | 50.9 | (11.4) | 49.6 | (12.6) |
|  | 36 months | 52.1 | (10.5) | 51.2 | (11.2) |
| Personal-social | |  |  |  |  |
|  | 6 months | 34.5 | (14.9) | 33.7 | (15.0) |
|  | 12 months | 37.2 | (14.3) | 36.5 | (14.4) |
|  | 18 months | 48.0 | (9.9) | 46.4 | (10.8) |
|  | 24 months | 46.5 | (8.0) | 45.6 | (8.9) |
|  | 30 months | 50.4 | (9.8) | 48.9 | (10.6) |
|  | 36 months | 50.6 | (9.8) | 49.4 | (10.7) |

Data represent mean (SD).

Abbreviations: J-ASQ-3, the Japanese translation of the Ages and Stages Questionnaires, Third Edition; SD, standard deviation.

**Table S3.** Comparison of numbers and proportions of neurodevelopmental delay in each domain of the J-ASQ-3 between participants included in and excluded from this study

|  | | Included in this analysis  (n = 42,172) | | Excluded from this analysis  (n = 31,658) | |
| --- | --- | --- | --- | --- | --- |
| Communication | |  |  |  |  |
|  | 6 months | 241 | (0.6) | 121 | (0.5) |
|  | 12 months | 44 | (0.1) | 16 | (0.1) |
|  | 18 months | 860 | (2.0) | 281 | (1.8) |
|  | 24 months | 1,524 | (3.6) | 573 | (3.5) |
|  | 30 months | 1,893 | (4.5) | 700 | (4.6) |
|  | 36 months | 1,548 | (3.7) | 552 | (3.3) |
| Gross motor | |  |  |  |  |
|  | 6 months | 4,203 | (10.0) | 1,880 | (8.5) |
|  | 12 months | 2,215 | (5.3) | 854 | (4.5) |
|  | 18 months | 1,763 | (4.2) | 604 | (3.9) |
|  | 24 months | 2,246 | (5.3) | 804 | (4.9) |
|  | 30 months | 1,620 | (3.8) | 564 | (3.7) |
|  | 36 months | 1,678 | (4.0) | 621 | (3.8) |
| Fine motor | |  |  |  |  |
|  | 6 months | 2,070 | (4.9) | 840 | (3.8) |
|  | 12 months | 2,241 | (5.3) | 975 | (5.2) |
|  | 18 months | 1,724 | (4.1) | 547 | (3.6) |
|  | 24 months | 807 | (1.9) | 301 | (1.8) |
|  | 30 months | 2,340 | (5.5) | 778 | (5.2) |
|  | 36 months | 2,987 | (7.1) | 1,096 | (6.7) |
| Problem solving | |  |  |  |  |
|  | 6 months | 4,404 | (10.4) | 1,892 | (8.6) |
|  | 12 months | 2,094 | (5.0) | 819 | (4.4) |
|  | 18 months | 1,649 | (3.9) | 495 | (3.3) |
|  | 24 months | 1,640 | (3.9) | 618 | (3.8) |
|  | 30 months | 2,242 | (5.3) | 760 | (5.1) |
|  | 36 months | 2,936 | (7.0) | 1,045 | (6.5) |
| Personal-social | |  |  |  |  |
|  | 6 months | 1,456 | (3.5) | 652 | (3.0) |
|  | 12 months | 464 | (1.1) | 184 | (1.0) |
|  | 18 months | 976 | (2.3) | 302 | (2.0) |
|  | 24 months | 1,063 | (2.5) | 400 | (2.4) |
|  | 30 months | 1,319 | (3.1) | 427 | (2.8) |
|  | 36 months | 1,256 | (3.0) | 434 | (2.6) |

Data represent n (%)

Cut-off scores for Japanese children^25^ were used.

Abbreviations: J-ASQ-3, the Japanese translation of the Ages and Stages Questionnaires, Third Edition.

**Table S4.** Adjusted odds ratios for neurodevelopmental delay in each domain of the J-ASQ-3 among children born to mothers who received epidural analgesia during labor, from 6 to 36 months old, without multiple imputation method

|  | Communication | |  | Gross motor | |  | Fine motor | |  | Problem solving | |  | Personal-social | |
| --- | --- | --- | --- | --- | --- | --- | --- | --- | --- | --- | --- | --- | --- | --- |
|  | aOR | (95%CI) |  | aOR | (95%CI) |  | aOR | (95%CI) |  | aOR | (95%CI) |  | aOR | (95%CI) |
| 6 months | 1.79 | (0.85, 3.74) |  | 1.27 | (1.02, 1.59) |  | 1.23 | (0.90, 1.69) |  | 1.25 | (1.01, 1.55) |  | 1.09 | (0.74, 1.61) |
| 12 months | 1.91 | (0.43, 8.43) |  | 1.10 | (0.81, 1.50) |  | 1.18 | (0.88, 1.60) |  | 1.08 | (0.79, 1.48) |  | 2.19 | (1.31, 3.68) |
| 18 months | 1.22 | (0.79, 1.89) |  | 1.39 | (1.02, 1.88) |  | 1.61 | (1.19, 2.16) |  | 1.38 | (1.00, 1.90) |  | 1.52 | (1.02, 2.25) |
| 24 months | 1.37 | (0.98, 1.91) |  | 1.26 | (0.94, 1.68) |  | 1.20 | (0.74, 1.92) |  | 1.39 | (1.01, 1.91) |  | 1.73 | (1.21, 2.49) |
| 30 months | 0.96 | (0.68, 1.37) |  | 1.08 | (0.76, 1.53) |  | 1.17 | (0.87, 1.58) |  | 1.22 | (0.91, 1.63) |  | 1.21 | (0.84, 1.75) |
| 36 months | 1.19 | (0.83, 1.70) |  | 0.91 | (0.62, 1.31) |  | 1.03 | (0.78, 1.37) |  | 1.18 | (0.90, 1.53) |  | 1.17 | (0.79, 1.72) |

Adjusted for maternal age at delivery, parity, delivery mode, duration of labor, comorbidity (diabetes and hypertension), body mass index before pregnancy, occupation in the first trimester, smoking status, alcohol consumption, educational level, annual household income, marital status at 6 months after childbirth, child’s sex, birthweight, Apgar score at 5 min after birth, feeding methods in infancy, nursery attendance, screen time at 12 months old, siblings cohabiting with the child, and study area.

Abbreviations: aOR, adjusted odds ratio; CI, confidence interval; J-ASQ-3, the Japanese translation of the Ages and Stages Questionnaires, Third Edition.

**Table S5.** Adjusted odds ratios for neurodevelopmental delay in each domain of the J-ASQ-3 among children born to mothers who received epidural analgesia during labor, from 6 to 36 months old, stratified by parity

|  | Communication | |  | Gross motor | |  | Fine motor | |  | Problem solving | |  | Personal-social | |
| --- | --- | --- | --- | --- | --- | --- | --- | --- | --- | --- | --- | --- | --- | --- |
|  | aOR | (95%CI) |  | aOR | (95%CI) |  | aOR | (95%CI) |  | aOR | (95%CI) |  | aOR | (95%CI) |
| Primiparas (n = 17,914) | | |  |  |  |  |  |  |  |  |  |  |  |  |
| 6 months | 3.04 | (1.23, 7.54) |  | 1.28 | (0.96, 1.71) |  | 1.23 | (0.78, 1.92) |  | 1.36 | (1.02, 1.80) |  | 0.95 | (0.52, 1.72) |
| 12 months | 3.28 | (0.67, 16.1) |  | 1.09 | (0.75, 1.58) |  | 0.96 | (0.63, 1.48) |  | 1.20 | (0.83, 1.72) |  | 3.20 | (1.59, 6.41) |
| 18 months | 1.13 | (0.68, 1.87) |  | 1.27 | (0.89, 1.81) |  | 1.33 | (0.90, 1.97) |  | 1.00 | (0.66, 1.52) |  | 1.59 | (1.00, 2.52) |
| 24 months | 1.11 | (0.72, 1.72) |  | 1.19 | (0.85, 1.67) |  | 1.40 | (0.81, 2.41) |  | 1.28 | (0.88, 1.84) |  | 1.93 | (1.30, 2.88) |
| 30 months | 0.87 | (0.55, 1.36) |  | 1.06 | (0.71, 1.58) |  | 1.18 | (0.82, 1.70) |  | 1.19 | (0.83, 1.69) |  | 1.34 | (0.88, 2.02) |
| 36 months | 1.31 | (0.84, 2.03) |  | 0.82 | (0.52, 1.29) |  | 0.98 | (0.70, 1.38) |  | 1.13 | (0.81, 1.58) |  | 0.95 | (0.57, 1.57) |
| Multiparas (n = 23,262) | | |  |  |  |  |  |  |  |  |  |  |  |  |
| 6 months | 1.37 | (0.49, 3.82) |  | 1.19 | (0.88, 1.60) |  | 1.06 | (0.71, 1.59) |  | 1.17 | (0.88, 1.56) |  | 1.10 | (0.69, 1.75) |
| 12 months | –* |  |  | 1.14 | (0.75, 1.74) |  | 1.14 | (0.77, 1.71) |  | 0.96 | (0.60, 1.55) |  | 1.11 | (0.51, 2.42) |
| 18 months | 1.44 | (0.74, 2.81) |  | 1.49 | (0.94, 2.36) |  | 1.65 | (1.10, 2.48) |  | 1.60 | (1.00, 2.56) |  | 0.86 | (0.42, 1.78) |
| 24 months | 1.55 | (0.99, 2.44) |  | 1.56 | (1.04, 2.33) |  | 0.95 | (0.46, 1.96) |  | 1.44 | (0.88, 2.37) |  | 1.36 | (0.74, 2.49) |
| 30 months | 1.33 | (0.86, 2.05) |  | 1.13 | (0.66, 1.93) |  | 1.12 | (0.74, 1.69) |  | 1.19 | (0.78, 1.83) |  | 1.17 | (0.67, 2.06) |
| 36 months | 1.38 | (0.88, 2.16) |  | 1.22 | (0.75, 1.96) |  | 1.14 | (0.77, 1.69) |  | 1.34 | (0.94, 1.89) |  | 1.49 | (0.87, 2.53) |

Adjusted for maternal age at delivery, delivery mode, duration of labor, comorbidity (diabetes and hypertension), body mass index before pregnancy, occupation in the first trimester, smoking status, alcohol consumption, educational level, annual household income, marital status at 6 months after childbirth, child’s sex, birth weight, Apgar score at 5 min after birth, feeding methods in infancy, nursery attendance, screen time at 12 months old, and study area. A multiple imputation method was used to reduce potential selection bias from missing variables.

* For communication delay at 12 months in multiparas, aOR could not be calculated due to the small number of the subjects.

Abbreviations: aOR, adjusted odds ratio; CI, confidence interval; J-ASQ-3, the Japanese translation of the Ages and Stages Questionnaires, Third Edition.

**Table S6.** Adjusted odds ratios for the incidence of neurodevelopmental delay in each domain of the J-ASQ-3 among children born to mothers who received epidural analgesia during labor, every 6 months after 18 months old, without multiple imputation method

|  | Communication | |  | Gross motor | |  | Fine motor | |  | Problem solving | |  | Personal-social | |
| --- | --- | --- | --- | --- | --- | --- | --- | --- | --- | --- | --- | --- | --- | --- |
|  | aOR | (95%CI) |  | aOR | (95%CI) |  | aOR | (95%CI) |  | aOR | (95%CI) |  | aOR | (95%CI) |
| 18-24 months | 1.32 | (0.88, 1.99) |  | 1.35 | (1.02, 1.78) |  | 1.23 | (0.68, 2.23) |  | 1.56 | (1.09, 2.24) |  | 1.82 | (1.18, 2.80) |
| 24-30 months | 0.93 | (0.55, 1.58) |  | 0.91 | (0.53, 1.58) |  | 1.21 | (0.85, 1.71) |  | 1.35 | (0.95, 1.91) |  | 1.18 | (0.73, 1.91) |
| 30-36 months | 1.43 | (1.00, 2.03) |  | 0.99 | (0.57, 1.71) |  | 0.98 | (0.67, 1.45) |  | 1.30 | (0.90, 1.88) |  | 1.26 | (0.69, 2.28) |

Adjusted for maternal age at delivery, parity, delivery mode, duration of labor, comorbidity (diabetes and hypertension), body mass index before pregnancy, occupation in the first trimester, smoking status, alcohol consumption, educational level, annual household income, marital status at 6 months after childbirth, child’s sex, birthweight, Apgar score at 5 min after birth, feeding methods in infancy, nursery attendance, screen time at 12 months old, siblings living together with the child, and study area.

Abbreviations: aOR, adjusted odds ratio; CI, confidence interval; J-ASQ-3, the Japanese translation of the Ages and Stages Questionnaires, Third Edition.

**Table S7.** Adjusted odds ratios for the incidence of neurodevelopmental delay in each domain of the J-ASQ-3 among children born to mothers who received epidural analgesia during labor, every 6 months after 18 months old, stratified by parity

|  | Communication | |  | Gross motor | |  | Fine motor | |  | Problem solving | |  | Personal-social | |
| --- | --- | --- | --- | --- | --- | --- | --- | --- | --- | --- | --- | --- | --- | --- |
|  | aOR | (95%CI) |  | aOR | (95%CI) |  | aOR | (95%CI) |  | aOR | (95%CI) |  | aOR | (95%CI) |
| Primiparas (n = 17,448) | | |  |  |  |  |  |  |  |  |  |  |  |  |
| 18-24 months | 1.04 | (0.60, 1.83) |  | 1.18 | (0.78, 1.77) |  | 1.26 | (0.60, 2.63) |  | 1.43 | (0.95, 2.17) |  | 2.03 | (1.25, 3.24) |
| 24-30 months | 1.05 | (0.55, 2.03) |  | 0.99 | (0.54, 1.81) |  | 1.22 | (0.80, 1.86) |  | 1.29 | (0.84, 1.99) |  | 1.31 | (0.75, 2.27) |
| 30-36 months | 1.81 | (0.76, 4.29) |  | 0.84 | (0.41, 1.74) |  | 0.97 | (0.62, 1.52) |  | 1.32 | (0.81, 2.14) |  | 0.65 | (0.24, 1.78) |
| Multiparas (n = 22,892) | | |  |  |  |  |  |  |  |  |  |  |  |  |
| 18-24 months | 1.53 | (0.90, 2.59) |  | 1.36 | (0.81, 2.28) |  | 1.19 | (0.52, 2.75) |  | 1.55 | (0.88, 2.72) |  | 1.91 | (1.01, 3.62) |
| 24-30 months | 1.06 | (0.55, 2.04) |  | 0.70 | (0.26, 1.93) |  | 1.14 | (0.70, 1.85) |  | 1.21 | (0.72, 2.01) |  | 1.13 | (0.56, 2.28) |
| 30-36 months | 1.33 | (0.57, 3.09) |  | 1.11 | (0.54, 2.30) |  | 1.01 | (0.58, 1.76) |  | 1.22 | (0.75, 1.97) |  | 1.65 | (0.78, 3.50) |

Adjusted for maternal age at delivery, mode of delivery, duration of labor, comorbidity (diabetes and hypertension), body mass index before pregnancy, occupation in the first trimester, smoking status, alcohol consumption, education, annual household income, and marital status at 6 months after childbirth, sex of the child, birthweight, Apgar score at 5 min after birth, feeding methods in infancy, nursery attendance, screen time at 12 months old, and study area. A multiple imputation method was used to reduce potential selection bias from missing variables.

Abbreviations: aOR, adjusted odds ratio; CI, confidence interval; J-ASQ-3, Japanese translation of the Ages and Stages Questionnaires, Third Edition.
